# Editorial: Aging and the social-emotional brain

**DOI:** 10.3389/fnagi.2024.1417410

**Published:** 2024-05-10

**Authors:** Edgar Soria-Gómez, Joaquín Piriz, Ignacio Torres-Alemán

**Affiliations:** ^1^Department of Neurosciences, University of the Basque Country UPV/EHU, Leioa, Spain; ^2^Achucarro Basque Center for Neuroscience, Science Park of the UPV/EHU, Leioa, Spain; ^3^IKERBASQUE, Basque Foundation for Science, Bilbao, Spain

**Keywords:** aging, delirium, cortex, emotion, statin, loneliness

In modern society, life expectancy increases year after year due in part to medical and scientific advances. However, at the same time, other pathological conditions appear in elders, affecting the general quality of life. This conundrum makes studying aging a crucial priority at many different levels, from economics to biology. This Research Topic reflects the scientific community's efforts, basic and applied, to understand specific traits associated to aging and its related pathologies, tackling different factors to get early detection of age-related diseases. Emotional balance is essential for healthy aging, and different stress-related factors could negatively influence this process. During our lifetime, we experience emotional challenges that mark, for good or for bad, our development and aging. For example, losing a romantic partner or a relative could trigger a cascade of events, resulting in social self-isolation and potentially a depressive episode. Thus, it is crucial to be able to detect those signs at an early stage. In this sense, Díaz-Mardomingo et al. showed a sex-dependent bias, being the males more affected, in how older adults perceive loneliness and its corresponding association with levels of cortisol, which could reflect a biological difference in stress-coping mechanisms between males and females. It remains to be investigated which areas of the brain are targeted by stress hormones associated with loneliness. An obvious candidate is the prefrontal cortex, one of the main brain regions regulating emotional valence and, therefore, implicated in stress-coping behaviors. Febo et al. decided to evaluate behavioral and functional differences in connectivity between young and older mice. Using protocols to study innate and learned emotional behaviors they showed that older mice might process emotional value differently, at least the negative ones because the authors did not find differences in appetitive behaviors (i.e., sucrose consumption). At this point, it is too early to characterize it as an affective deficit. Such differences could reflect behavioral adaptations necessary to cope with stressful events. Younger mice display greater locomotor activity in novel environments, and social exploration could reflect faster stimuli processing than aged mice. Likewise, learned-fear expression is also modified in elder mice, that display higher freezing responses. Interestingly, such age-dependent behavioral changes could be the result of altered functional and structural connectivity between frontal cortical areas and subcortical regions like the hippocampus. It would be interesting to analyze if the changes in connectivity are similar in a “loneliness” mice model.

Besides psychological and social factors, other conditions during aging can precipitate affective disturbances. Among them, vascular illnesses have been shown to be associated with depressive episodes: Vascular Depression (VaD). In fact, several hypotheses point out that disruption in cerebral connectivity could underlie the cognitive and emotional impairments present in VaD. In an effort to fully understand VaD and to identify biological markers for the early detection of this condition, Lan et al. performed a proteomic biomarker study in patients with VaD that could help with the development of protein-based models using machine-learning methods. Their results showed that pathways involved in synaptic events and axon guidance could affect VaD. Moreover, other proteins, such as dioxygenase and ubiquitins, were identified as potential biomarkers of VaD. Undoubtedly, more efforts trying to functionally associate peripheral and central processes will be extremely useful to understanding the pathophysiology of emotion and cognition. As part of this endeavor, Xia et al. reported a potential link between congestive heart failure and resulting delirium episodes using a retrospective analysis. Moreover, several studies show that statins could reduce all-cause mortality. In this case, the authors focused on the role of statins as protectors against delirium. Statins are common drugs used mainly to control cholesterol and triglycerides. Therefore, this study opens new viewpoints on how a treatment used for a certain condition can benefit others. In particular, this retrospective study confirmed the positive effect of statins, potentially reducing the appearance of delirium. Delirium episodes are more common in older adults but are induced by specific, acute illness or injury, such as post-surgical complications. Therefore, as in the case of statins, it is of great importance to count on as many prevention/therapeutic tools as possible. In this case, Zhu et al. decided to perform a pragmatic study to revise the use of s-ketamine, an anesthetic drug that has been shown to have rapid antidepressant effects, as a potential treatment for postoperative delirium. This type of study is of great value and hopefully will result in a better understanding of the complexity of age-related pathologies, which will ultimately impact our daily lives.

## Author contributions

ES-G: Conceptualization, Writing – original draft, Writing – review & editing. JP: Writing – review & editing. IT-A: Writing – review & editing.

